# Anatomical implant region – a critical determinant for the osteogenic potency of small extracellular vesicles and rhBMP-2

**DOI:** 10.1007/s00068-025-03036-w

**Published:** 2025-12-18

**Authors:** Christian Deininger, Andrea Wagner, Florian Wichlas, Eva Rohde, Thomas Freude, Amelie Deluca, Johann Fierlbeck, Martin Holzleitner, Sascha Senck, Herbert Tempfer, Mario Gimona, Andreas Traweger

**Affiliations:** 1 Institute of Tendon and Bone Regeneration, Paracelsus Medical University, Salzburg, Austria; 2 Department of Orthopaedics and Trauma Surgery, Medical University Graz, Graz, Austria; 3 Austrian Cluster for Tissue Regeneration, Vienna, Austria; 4 Department of Orthopedics and Traumatology, Salzburg University Hospital, Paracelsus Medical University, Salzburg, Austria; 5 Department of Transfusion Medicine, Salzburg University Hospital, Paracelsus Medical University, Salzburg, Austria; 6 GMP Unit, Paracelsus Medical University, Salzburg, Austria; 7 Department of Technology Transfer, Paracelsus Medical University Salzburg, Salzburg, Austria; 8 Computed Tomography Research Group, University of Applied Sciences Upper Austria, Wels, Austria; 9 Research Program “Nanovesicular Therapies”, Paracelsus Medical University, Salzburg, Austria

**Keywords:** Extracellular vesicles, Exosomes, Osseointegration, BMP-2

## Abstract

**Purpose:**

Mechanical stabilization is crucial for bone healing, yet complex fractures, particularly osteoporotic or comminuted, remain challenging due to impaired implant osseointegration, resulting in implant loosening or non-unions. This study investigated whether co-application of small extracellular vesicles (sEVs) derived from human umbilical cord mesenchymal stromal cells (hUC-MSC-sEVs) with a low dose of recombinant human bone morphogenetic protein 2 (rhBMP-2) could enhance screw implant osseointegration.

**Methods:**

A novel small animal model was established to evaluate the effect of anatomical femur regions on screw integration. Six weeks postoperatively, bone formation and bone–implant contact were assessed by micro-computed tomography and descriptive histology. Biomechanical stability was determined using pull-out tests.

**Results:**

Outcomes differed significantly between the proximal and distal implant locations, with no improvements in osseointegration observed in the distal region. In the proximal region, application of hUC-MSC-sEVs alone did not significantly improve osseointegration, whereas local application of 1.5 µg rhBMP-2 resulted in measurable biomechanical improvements. No additive or synergistic effects were observed when sEVs were co-administered with rhBMP-2. Descriptive histology supported these findings, demonstrating the most pronounced bone formation at the proximal site following rhBMP-2 treatment.

**Conclusion:**

hUC-MSC-sEVs did not enhance screw implant osseointegration and slightly reduced new bone formation. In contrast, a low dose of rhBMP-2 (1.5 µg) promoted implant integration, with no additive effect when combined with sEVs. Notably, the osteogenic effect of rhBMP-2 was observed only at the proximal femoral site, indicating that anatomical location critically influences implant osseointegration. These findings highlight the importance of considering anatomical region when evaluating osteoinductive treatments and implant materials in small animal models.

**Supplementary Information:**

The online version contains supplementary material available at 10.1007/s00068-025-03036-w.

## Introduction

One of the most critical factors for successful fracture healing is adequate mechanical stability of the fracture fixation [1]. However, the mechanical performance of fracture fixation is strongly affected by the morphometric, geometric and biomechanical properties of the host bone as well as appropriate vascular supply. Although modern osteosynthesis implants have demonstrated to provide increased mechanical stability in experimental analyses, this does not necessarily translate to improved stabilization of clinically demanding fractures [2, 3]. The treatment of, e. g. osteoporotic fractures is not yet satisfactorily resolved and contributes to significant morbidity or mortality rates [3, 4]. The achievable stability is heavily influenced by the type of fracture. Complex comminuted fractures, where the bone fragments contribute minimally to stability, pose a particular challenge [5]. In such cases, the chosen implant by itself must provide primary stability [6, 7]. The development of angular stable implants, such as plates and nail osteosyntheses, resulted in a significant improvement of primary stability after osteosynthesis [8–13]. The angular stable screws used in modern plate osteosynthesis achieve this stability by securing stable fixation at three points: the transcortex, the ciscortex and the plate itself. This usually results in sufficient stability and complication-free healing of a closed fracture in a healthy patient [14]. In complex comminuted fractures, achieving adequate stability is generally more difficult and loosening of the osteosynthesis material can be a consequence of this insufficient stability. To increase the primary stability of an osteosynthesis, adequate osseointegration of the implanted material is imperative [15]. However, most orthopedic implants lack intrinsic osteoinductivity or osteoconductivity and mainly address pure mechanical repair. A possible solution to this complication is to promote or accelerate the osseointegration of the screws via functionalization with osteoinductive agents [4]. The first growth factor approved by the Food and Drug Administration (FDA) for clinical use to promote bone healing was recombinant human bone morphogenetic protein 2 (rhBMP-2; InFuse®) [16]. However, local and systemic adverse events due to the use of excessively high dosages has been reported, resulting in unfavorable clinical outcomes [17, 18]. As a result, there is significant interest in developing strategies to reduce the required amount of BMP-2 while maintaining its efficacy [19].

At the implant-tissue interface a coordinated interplay between various cell types is required for full and solid integration. Therefore, next to the delivery of growth factors, the use of mesenchymal stromal cells (MSCs) has demonstrated beneficial effects to enhance bone repair. However, routine clinical application of stem cell-based therapies is still hampered, in part due to safety concerns relating to the transplantation of actively dividing cells [20]. As it has become increasingly clear that the therapeutic potency observed after administration of MSCs is primarily conveyed via their bioactive secretome, small extracellular vesicles (sEVs) harvested from various stem cell sources have been investigated for their bone regeneration capacity in general [21], and in particular to improve osseointegration of implants [22, 23]. Overall, bone healing is a multifactorial process [24, 25]. Not surprisingly, a combination of different osteogenic agents show an additive effect in some studies [26]. For example, it has been shown that in a rat critical-sized defect model, a significant increase in newly formed bone can be achieved by the combination of a coating with hydroxyapatite and rhBMP-2 compared to the administration of each substance alone [27]. Additionally, we recently demonstrated that combining small extracellular vesicles (sEVs) derived from human umbilical cord mesenchymal stem cells (hUC-MSC-sEVs) with a low dose of rhBMP-2 significantly enhanced the regeneration of a segmental metaphyseal defect in osteoporotic bone, compared to the use of either treatment alone [28]. The primary aim of this study was to assess the potential of hUC-MSC-sEVs, in combination with a low dose of rhBMP-2, to enhance implant osseointegration in a small animal model. Additionally, to evaluate the influence of the anatomical implant region, we developed a novel surgical model that simultaneously allows assessment of screw osseointegration in both the proximal and distal metaphysis of the rat femur. A list of abbreviations can be seen in Table 1.

**Table 1:** List of abbreviations

Abbreviation	Meaning	Abbreviation	Meaning
BMP-2	Bone morphogenetic protein-2	sEVs	small extracellular vesicles
hUC-MSC	human umbilical cord mesenchymal stromal cells	µCT	micro-computed tomography
FDA	Food and Drug Administration	MSCs	mesenchymal stromal cells
TFF	tangential flow filtration	NTA	nanoparticle tracking
s. c	subcutaneous	Alg	alginate
a. p	anteroposterior	PFA	Paraformaldehyde
VS	voxel size	BV/TV	Bone Volume/Tissue Volume
BIR	Bone Intersection Ratio	ROI	region of Interest
GV	grey value	NTA	nanoparticle tracking analysis
MCSP	melanoma-associated chondroitin sulfate proteoglycan	Fmax	maximum pull-out force
EVs	Extracellular vesicles	bone marrow MSCs	bMSCs
HA	hyaluronic acid	PCL	Polycaprolactone
ARRIVE	Animal Research: Reporting of In Vivo Experiments	N	Newton
N/mm	Newtons per millimeter		

## Methods

### Purification and characterization of hUC-MSC-sEVs

hUC-MSC-sEVs were prepared as previously described [28–30]. Briefly, UC-MSCs used in this study were derived from a single donor and from the primary expanded MSCs master and working cell banks were established. hUC-MSCs were expanded in a fibrinogen-depleted culture medium (α-MEM supplemented with 10% v/v pooled human platelet lysate and 5.5 mg/mL dipeptiven; Merck, Vienna, Austria) and subsequently, upon reaching 60–70% confluency the growth medium was exchanged with vesicle-depleted harvest medium (α-MEM supplemented with 2.5% v/v pooled human platelet lysate, 5.5 mg/mL dipeptiven). The tri-lineage differentiation capacity of the hUC-MSC has been previously demonstrated [30]. Vesicle depletion of culture media was performed by tangential flow filtration (TFF) using a 750 kDa hollow fiber filter (Spectrum Labs-Repligen, Breda, The Netherlands). Cells were cultured for a total of 24 h and culture supernatants were collected, centrifuged for removal of cell debris (2.500 × g, 20 min), and passed through a 0.22 μm filter (Merck, Vienna, Austria). Processed culture media were then further reduced by TFF (100 kDa hollow fiber filter, Spectrum Labs-Repligen, Breda, The Netherlands) and buffer-exchanged into sterile 0.9% NaCl by diafiltration. Finally, hUC-MSC-sEVs were collected by ultracentrifugation at 120 000 × g for 3 h at 18 °C, and the resulting pellets were re-suspended in an appropriate volume of Ringer’s lactate to achieve a final particle concentration of 2 × 10^9^ in 15 µl (single dose), again sterile filtered (0.22 μm) and stored in glass vials at -80 °C until further use.

Particle concentration and size distribution of the EV preparation was assessed by nanoparticle tracking analysis in light-scatter mode (ZetaView PMX-110, Particle Metrix). An aliquot of the frozen EV preparation was thawed and diluted in PBS to a working concentration of 4–7 × 10⁷ particles/mL. The instrument was calibrated with 100 nm yellow/green-fluorescent polystyrene standard beads prior to each run. Acquisition settings were as follows: minimum brightness 20 AU, temperature 21.5 °C, shutter 70 AU, sensitivity 85 AU. For each sample, two technical recordings were acquired across 11 measurement positions. Particle size distributions were computed from the tracked trajectories based on the Stokes–Einstein equation using the ZetaView software (PMX-110, Version 8.4.2) and particle size is reported as mean ± SD based on triplicate measurements. Total protein content was determined by fluorescence spectroscopy (Qubit 3.0; Life Technologies; Vienna Austria).

The surface antigen profile of the hUC-MSC-sEV preparation was assessed in duplicate using the MACSPlex Exosome Kit (Miltenyi Biotec) following the manufacturer’s instructions and an established SOP, using 5 × 10⁸ total particles as input. Measurements were acquired on a FACS Canto II flow cytometer (BD Biosciences). Signal intensities were normalized to the combined APC fluorescence of the tetraspanins CD9, CD63, and CD81.

### Ethical approvals

All animal experiments and procedures were conducted in accordance with Austrian laws on animal experimentation and were approved by Austrian regulatory authorities (Permit No. 220–0.466.197). The use of human UC-MSCs for sEV enrichment was approved by the Ethics Committee of the province of Salzburg (approval number 415-E/1776/4–2014) and with informed consent from the donor.

### Animal study design

42, three-month-old, female Sprague Dawley rats (*R. norwegicus;* Janvier Labs SAS, France) weighing between 250–300 g were randomly assigned to 4 intervention groups.

Thirty minutes preoperatively the animals received 0,03 mg/kg buprenorphin as a subcutaneous (s. c.) injection. For anesthetic induction the rats were placed in an airtight box, which was filled with 4–5 vol.% isoflurane in oxygen. To maintain anesthesia 2–3 vol.% isoflurane at an oxygen flow rate of 500 ml/min was administered using a mask. Before surgery, each animal received an antibiotic (Clindamycin, 45 mg/kg) and 1.8 ml Ringer´s solution s.c. During surgery all animals were warmed using an electric heating pad to prevent hypothermia (Harvard Apparatus, Holliston, MA, USA). After the induction of anesthesia, the hair in the surgical area was clipped and aseptic preparation for surgery was performed. The animals were placed on their backs, a lateral 3–4 cm skin incision was made and the shaft of the right femur was carefully exposed.

**Fig. 1:**
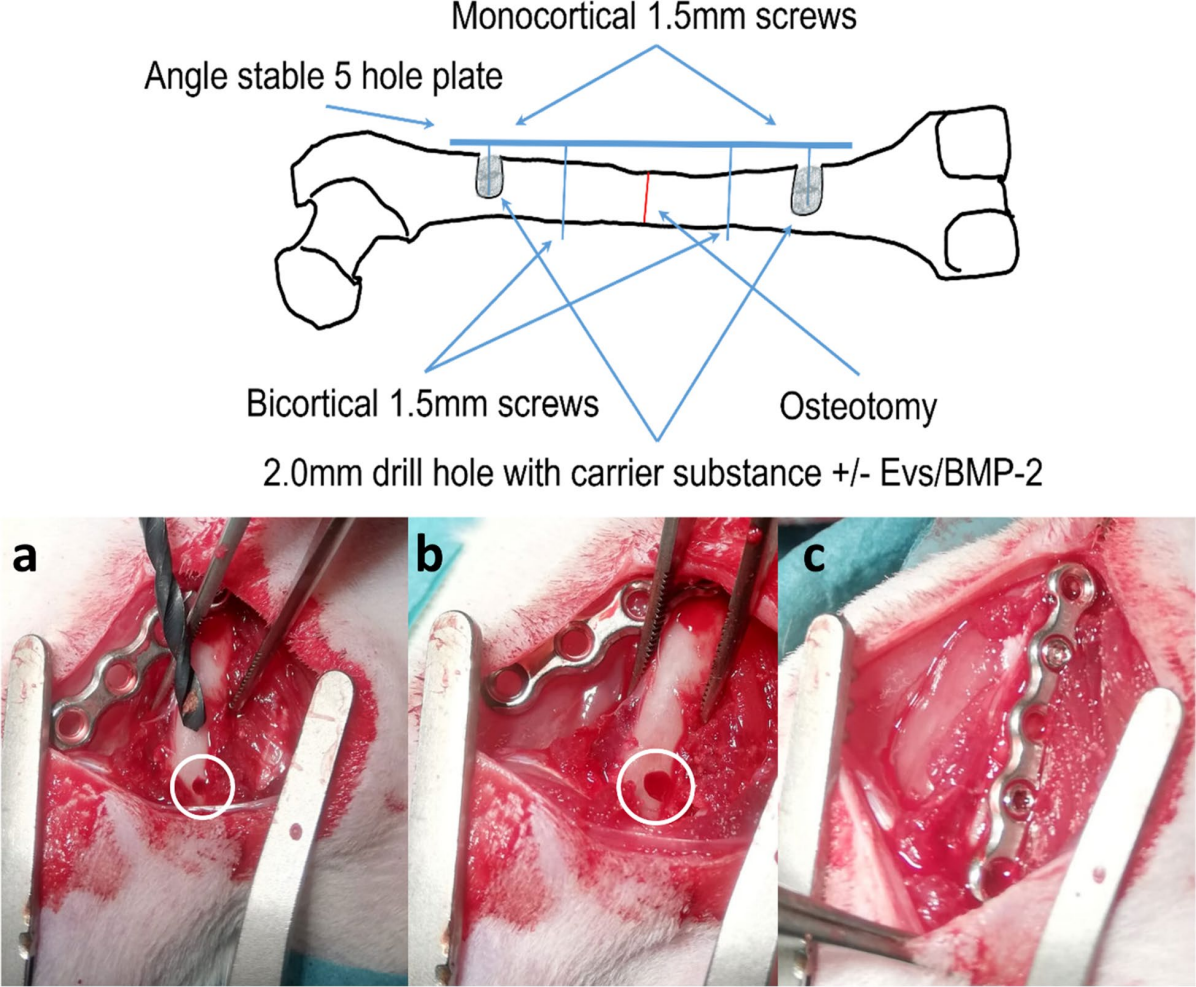
Schematic of the experimental setup. **a** Femur with angular stable plate and bicortical screws (blue), screw implant (red) in 2 mm drill hole loaded with loaded or un-loaded alginate hydrogels (grey) and a diaphyseal osteotomy (asterisk). **b** After drilling a 1.1 mm monocortical hole, **b** the implant region was widened to 2 mm using a drill bit and **c** after rotating back the locking plate, a shortened locking screw was inserted

Subsequently, the bicortical drilling of the first 1.1 mm drill hole was performed, using the second most proximal hole of an adapted five-hole plate as guide (1.5 mm LH Locking Plate, stainless, Veterinary Orthopedic Implants, Orly, France). In the second step, the angle-stable 5-hole plate was bicortically fixed with a locking screw (DT Locking Screw self-tap, stainless, 1.5 × 6.0 mm; Veterinary Orthopedic Implants, Orly, France) and aligned orthogradely along the femur diaphysis. Using a 1.1 mm drill, the 2 monocortical holes were drilled in the most proximal and distal plate holes, and the second bicortical hole was drilled in the second most distal plate hole. After slightly loosening the first screw, the plate was rotated by 40° and the monocortical drill holes were enlarged to 2.0 mm diameter with a drill bit. Subsequently, after thoroughly rinsing with sterile saline solution, alginate hydrogels were press-fit into the 2-mm drill defects. Alginate gels (35 µL final volume per defect) were prepared from sterile sodium alginate (PRONOVA SLG-20, 75–150 kDa; NovaMatrix, Sandvika, Norway) at a final concentration of 1.5 mg/mL. Gelation was induced by adding sterile CaCl₂ to a final concentration of 2 µg/mL, and samples were allowed to set at room temperature for 5–10 min. For volume adjustment and for preparation of the alginate-only controls, sterile NaCl was used. Four treatment groups were generated: (I) alginate only (Alg only), (II) alginate loaded with 2 × 10⁹ hUC-MSC-sEVs (Alg sEV), (III) alginate supplemented with 1.5 µg rhBMP-2 (Alg rhBMP-2; R&D Systems Inc., MN, USA), and (IV) alginate co-loaded with 2 × 10⁹ hUC-MSC-sEVs and 1.5 µg rhBMP-2 (Alg sEV rhBMP-2). The plate was then rotated back and the plate was fixed with bicortical screws (Fig. 1a-c). Subsequently, 2 shortened monocortical screws (DT Locking Screw self-tap, stainless, 1.5 × 4.0 mm; Veterinary Orthopedic Implants, Orly, France) were inserted into the most proximal and distal plate holes of the angular-stable plate. This resulted in the angle-stable implant not having a direct contact area with the bone, allowing in-growth of newly formed bone tissue (Fig. 1a-c). In the last step, to mimic a fracture and to introduce moderate instability, an osteotomy measuring 0.66 mm was performed in the center of the diaphysis using a Gigli wire saw (RISystem AG, Lanquart, Switzerland). The muscle was then sutured continuously over the plate and the skin was closed using surgical clips (Fine Science Tools, Heidelberg, Germany). Immediately after surgery, a control X-Ray in 2 planes was performed. As postoperative analgesia the animals received a daily dose of buprenorphine (0.03 mg/kg, twice daily) provided by subcutaneous injections for a total of 3 days and oral tramadol-hydrochloride (20 mg/kg body weight, once daily) via their drinking water for a total of 7 days. The animals had free access to food and water and were frequently monitored for any complications, weight loss or abnormal behavior. In the case of wound infection or irritation, animals were given 45 mg/kg Clindamycin s.c. Wound clips were removed after 7 days.

6 weeks postoperatively, the animals were euthanized by intracardial barbiturate injection under full anesthesia. The femurs were harvested for assessment of bone healing by biomechanical testing, histology and μCT analysis.

### Radiological follow-ups

The X-ray imaging was performed using the following equipment and settings:

X-ray Source: Orange 9020HF (EcoRay Co., Ltd.; Seoul, Korea), detector: Flat Panel Detector FPD2P(Venu1417P) (iRay Technology Ltd., Shanghai, China), Software: Conaxx2 VET (PROTEC GmbH & Co. KG, Oberstenfeld, Germany). The X-ray source was set to 44 kV and 20 mAs to ensure optimal imaging conditions. The Flat Panel Detector was used to capture high-resolution images, and the Conaxx2 VET software was employed for image acquisition and analysis.

X-rays were performed at 1, 2, 4, and 6 weeks postoperatively to evaluate bone healing and the status of the osteosynthesis material. While under general anesthesia (see above), the animal was positioned on its back, with the right hind limb extended using an elastic band and rotated to allow X-rays to be captured in both lateral and anteroposterior (a.p.) views.

### Microcomputed tomography

**Fig. 2:**
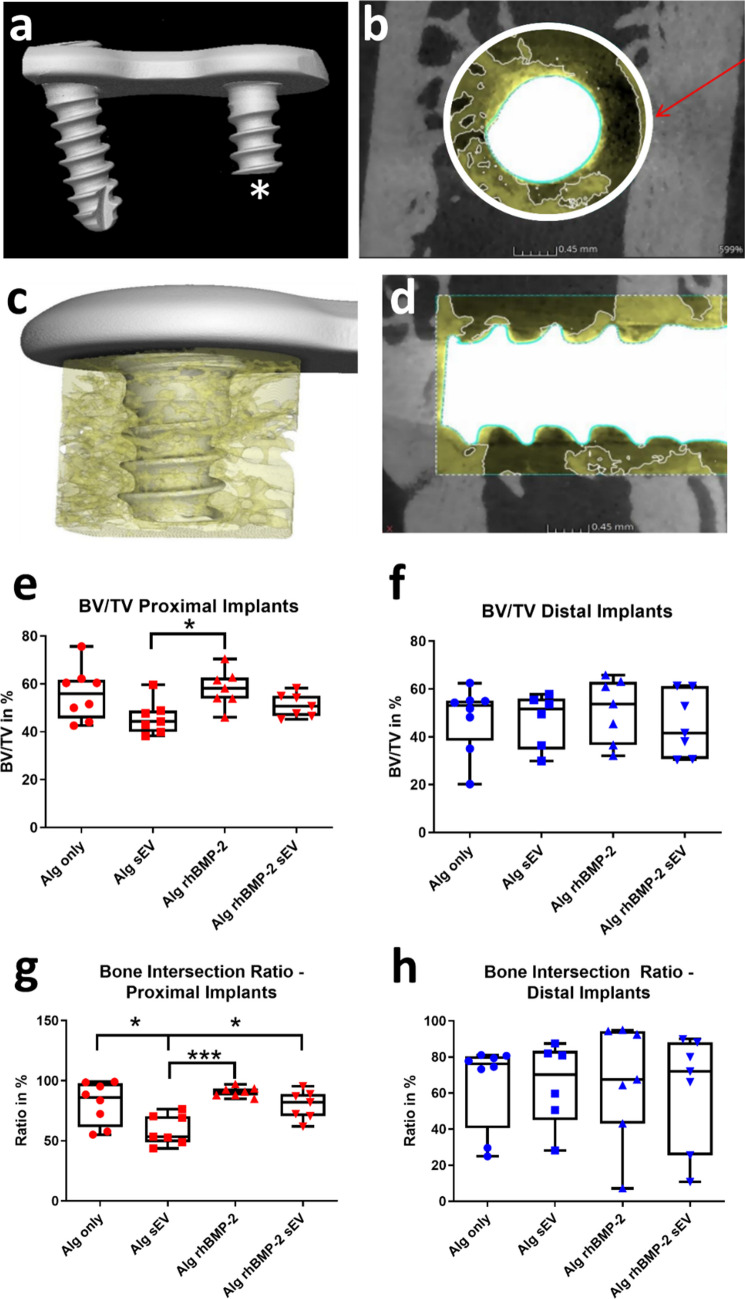
µCT evaluation of screw inmplant area. **a** Three-dimensional rendering of the angular stable plate with the bicortical screw and the monocortical implant to be tested (asterisk). **b**/**c** 2.2 mm diameter was set to determine the volume of the newly formed bone (yellow). **d** Contact surface area was determined to calculate total Bone Intersection Ratio (BIR) in 3D (green). BV/TV (%) values determined for **e** proximal and **f** distal implants. BIR area (%) calculated for **g** proximal and **h** distal anatomical regions. *N* = 7–8; *p < 0.05, ***p < 0.005

At 6 weeks after surgery animals were euthanized and femora were explanted, fixed in 4% paraformaldehyde (PFA) and samples from each treatment group (*n* = 7–8) were examined by microcomputed tomography (μCT) using a GE Phoenix Nanotom® S µCT system (General Electric, Boston, Massachusetts, USA). Each sample was stored in a sealed polymer sample tube to prevent dehydration during the µCT scanning procedure and samples were scanned using the following parameters: 150 kV tube voltage, 80 μA tube current, and an integration time of 1000 ms. All samples were scanned at a voxel size (VS) of 5 µm. A 0.25 mm physical tin filter-plate was applied to reduce beam hardening artefacts. The Bone Volume/Tissue Volume (BV/TV) within a 2.2 mm radius around the monocortical screws and the Bone Intersection Ratio (BIR) of the respective screw was determined (Fig. 2a -d). BV/TV was calculated and is expressed as mean ± SD (%). Image reconstruction of the acquired projection data was performed using X-AID (MITOS GmbH) involving a beam hardening correction [31]. Post processing was performed in VGSTUDIOMAX 3.5 (Volume Graphics GmbH). Volume data post-processing involved several evaluation steps that were repeated for all analyzed samples. Firstly, image data was aligned so that the X-, Y-, and Z-axes of the software coordinate system coincided with the frontal, sagittal, and transversal plane respectively. This was done for all samples in order to define a comparable, cylindrical region of Interest (ROI; diameter: 2.2 mm; height 3.5 mm) for all datasets. The central axis of the applied cylinder coincided with the longitudinal axis of the respective screw shaft. The bottom surface of the plate implant was chosen as landmark for defining the base of the ROI. Subsequently, the region growing tool provided by VGSTUDIOMAX 3.5 was utilized to segment the implant and the screws present in the ROI. This segmentation was then subtracted from the complete ROI in order to be eliminated from the rest of the evaluation. Afterwards, the remaining volume data was extracted and a median filter (kernel size: 3 × 3x3 voxel) was applied for the purpose of images denoising. For segmentation of newly formed bone, a grey value (GV) threshold was manually set to exclude the liquid phase within the sample and samples were manually curated to exclude beam hardening artefacts. Bone volume fraction, i.e. bone volume divided by total volume (BV/TV; in percent) was then computed in VGSTUDIOMAX 3.5. In addition, we quantified the bone-screw interface by computing the intersection between the surface of the segmented bone and the screw shaft in VGSTUDIOMAX 3.5. Firstly, an advanced surface determination was carried out to create a surface of the entire bone in the final ROI. Secondly, the region growing tool was utilized to establish an additional ROI only featuring the metal screw. This ROI was subsequently dilated by one voxel using the erode/dilate function in order to create an intersection between the surface of the screw and the bone surface. We defined the contact area of bone and screw by this intersection (in percent).

### Biomechanical testing

**Fig. 3:**
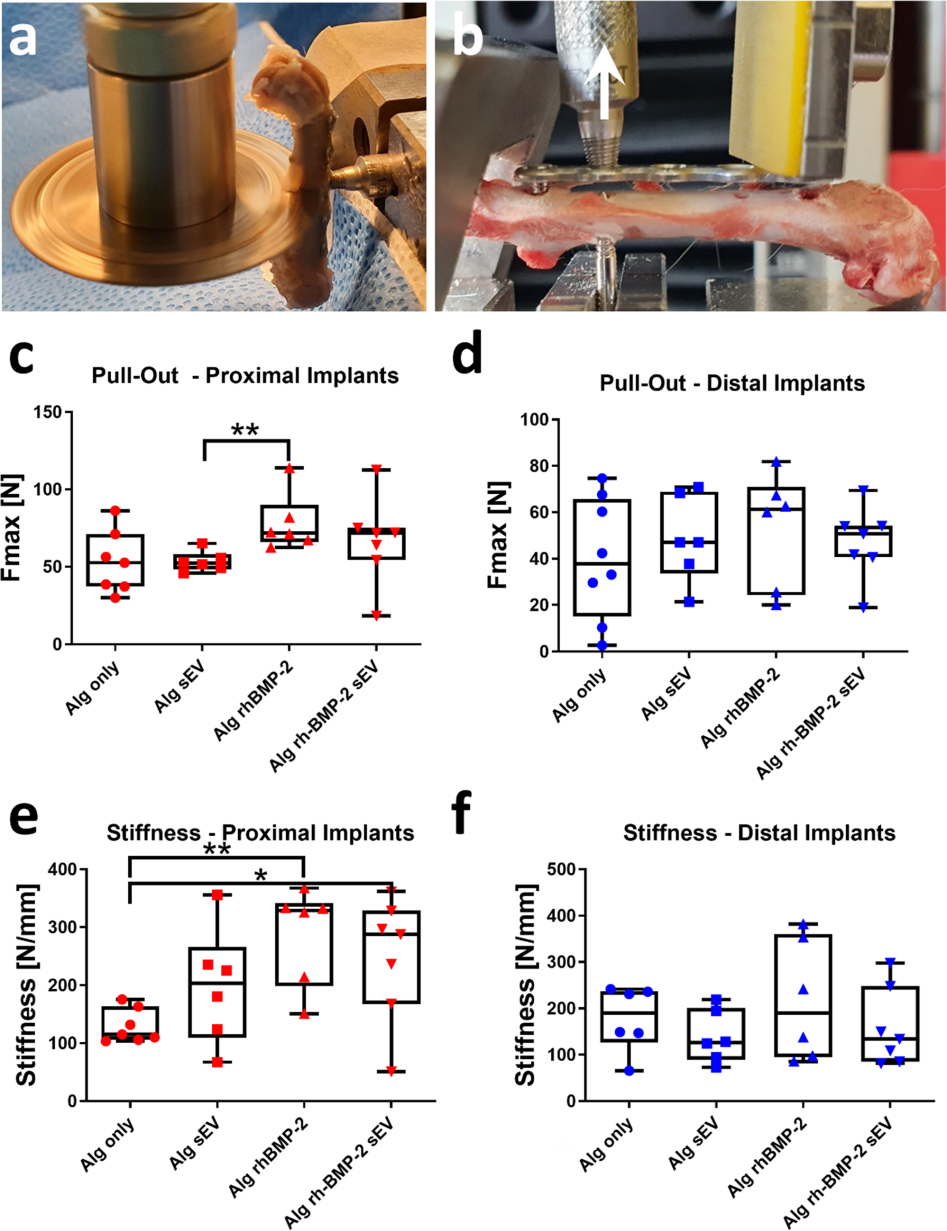
Biomechanical evaluation of screw implants. **a** Preparation of a full osteotomy prior to the pull-out test. The saw was adjusted to stop precisely at the level of the plate to avoid loosening of the screws due to implant vibration. **b** Pull-out test of a proximal implant. Arrow indicates the direction of tension applied. Samples were mounted onto a Kirschner wire using one of the bicortical drill holes and tension was applied to the plate using an interlocking drill guide. Maximum pull-out force (N) was separately determined for the **c** proximal and **d** distal implants. Significant increase in Fmax was only determined for proximal implants treated with rhBMP-2 compared to the sEV-treated group (*p* = 0.0043). Stiffness values (N/mm) calculated from the linear region of the force–elongation curves was significantly greater only in the proximal hole defects (**e**) in the rhBMP-2 group compared to Alg only (*p* = 0.0047) and in the rhBMP-2/sEV group compared to Alg only (*p* = 0.0379). **f** no significant differences were determined for any of the distal treatment groups. N = 6–7; *p < 0.05, **p < 0.01

After µCT scanning the explanted femora were prepared for a pull-out test. To test the 2 implants separately for each anatomical region, an osteotomy of the femur was centrally performed between the two bicortical screws. For this purpose, a custom-made device was used to ensure no contact with the osteosynthesis plate occurred in order to avoid screw loosening due to vibrations (see Fig. 3a). After complete osteotomy, the 2 bicortical screws were carefully removed and the specimens were clamped in a Zwick universal tensile testing machine (Zwick/Roell 500N Zwicki, Ulm/Einsingen, Germany). Samples were mounted onto a 1.6 mm Kirschner wire using the drill hole of a removed bicortical screw. An angle-stable drill bush was attached to the plate using the thread next to the screw to be evaluated and pull-out test was performed at a speed of 1 mm/sec and force/elongation curves were recorded. Proximal and distal implants were tested separately in a similar manner. Stiffness was calculated from the linear proportion of the force/elongation curve and is expressed as N/mm. The maximum force (Newton) for extraction of the screw was determined from the peak force recorded during testing.

### Descriptive histology

For histology, femurs were disarticulated (6 weeks post-surgery) at the hip and knee joints, and the majority of the muscle tissue was removed. After fixing samples in 4% paraformaldehyde (PFA) in PBS for 24 h, samples were then transferred to 2% PFA in PBS and stored at 4 °C until further processing. Three biological replicates of each treatment group were decalcified in 2% PFA/12.5% EDTA solution (pH = 7.5) and evaluated by histology. The progression of the decalcification process was monitored by regular X-ray examinations. After at least 8 weeks, specimens were prepared for histology and 7 µm sections were stained with Movat pentachrome stain. High-resolution digital images were acquired using a Zeiss Axioplan microscope equipped with an AxioCam MRc5 CCD camera (Carl Zeiss GmbH, Vienna, Austria).

### Statistical analysis

Quantitative µCT and biomechanical data were evaluated for significant differences using GraphPad Prism v. 9.02 (San Diego, CA, USA). All samples were tested for normal distribution using the Shapiro Wilk test. A one-way ANOVA test with post-hoc pairwise comparison (Tukey’s) was performed. For pairwise comparisons an unpaired t-test or Mann Whitney test was used. Significance was set at α = 0.05. Simple linear regression analyses were conducted to evaluate the relationship between µ-CT parameters and biomechanical outcomes in proximal and distal regions separately. Data were plotted, and a best-fit line was calculated using the least-squares method. Best fit values (R^2^), slopes, Y-intercepts, and p-values were determined for each dataset. Confidence intervals for the slope and Y-intercept were calculated to assess the precision of the estimates, and the statistical significance of the slope being different from zero was evaluated using an F-test.

## Results

### Characterization of hUC-MSC-sEVs

sEVs obtained from hUC-MSC-sEVs hUC-MSC were enriched according to the procedure detailed in the Methods section. Nanoparticle tracking analysis (NTA) revealed a mean particle diameter of 120.7 ± 1.7 nm (*n* = 3) and a mean particle concentration of 5.77 × 10^10^/ml. Total protein concentration determined by was 4.94 mg/ml and therefore the final preparation contained 1.168 × 10^10^ particles per milligram of total protein. Bead-based multiplex surface profiling confirmed the expression of the canconical EV tetraspanins CD9, CD63, and CD81 on the hUC-MSC-sEV preparation. In addition, markers typically associated with MSC-derived vesicles, including CD29 (integrin β1), CD44 (hyaluronic acid receptor), CD49e (integrin α5), and melanoma-associated chondroitin sulfate proteoglycan (MCSP), were robustly detected. In contrast, the platelet-associated markers CD41b, CD42a, and CD62P were not detected, indicating that the vesicle preparation was largely free of platelet-derived contaminants (see Supplemtary Fig. 1).

### General animal health

A total of 41 animals were included in the study for femoral analysis. One animal from the alginate-only treatment group was excluded due to misaligned drill hole placement affecting both implant sites. No major intraoperative complications were observed. Postoperatively, all animals exhibited normal behavior and resumed full weight-bearing within 24 h after surgery. Throughout the remainder of the study, no further exclusions were necessary due to weight loss or other postoperative complications. There were no obvious local signs of inflammation due to the local delivery of alginate loaded with rhBMP-2, sEVs, or in combination and the plate osteosynthesis was well tolerated.

### Surgical technique and radiographic evaluation

The aim of this newly established small animal model was to allow bone ingrowth after the delivery of osteoinductive agents (rhBMP-2, sEVs, or combination of both), to subsequently evaluate osseous integration of a screw implant at different anatomical regions of the rat femur. Therefore, we made use of mini-locking plates, allowing a stable positioning of the monocortical screw (tested implant) in a 2 mm drill hole defect as outlined in the methods section (see also Fig. 2a). For none of the animals, intraoperative complications, such as fractures or accidental bicortical drilling occurred and the stabilization of the 0.66 mm osteotomy with 2 bicortical angle-stable screws and plate osteosynthesis was sufficient to ensure safe postoperative mobilization of the animals. Routine radiographic examinations of the treated femora did not reveal any implant failure or loosening of the osteosynthesis material during the 6-week study period and at the endpoint, all osteotomies were fully consolidated as evidenced by radiography.

### Quantification of newly formed bone and bone intersection ratio by µCT

One sample of the following group was excluded due to improper positioning of the screw implant: sEV- treatment at the distal location.

The newly formed bone volume within the drill defect (BV/TV; Fig. 2b/c) and the resulting contact area with the screw implant (bone intersection area, BIR; Fig. 2d) was determined by µCT for the proximal and distal implantation sites. Interestingly, overall analysis of the different treatment groups for the proximal implants yielded differential results, whereas for the distal implants no significant differences where observed (Fig. 2e/f). For the proximal anatomical region BV/TV was significantly greater for the defects treated with rhBMP-2 (58.11% ± 7.82%) when compared to the sEV group (45.92% ± 7.16%; *p* = 0.0175). For none of the other pairwise comparison a significant difference was determined, with defects treated with alginate only yielding a BV/TV of 55.87% ± 10.95% and after treatment with a combination of sEV and rhBMP-2 a BV/TV of 51.08% ± 4.88%.

Statistical evaluation of BIR determined for the proximal implants (Fig. 2g) revealed a highly significant increase in contact area between the rhBMP-2 (90.72% ± 4.12%) and the sEV-treated group (59.30% ± 12.46%; *p* = 0.0006). Moreover, BIR was significantly higher in Alg only group (81.35% ± 17.68%) when compared to sEV group (59.30% ± 12.46%; *p* = 0.014). Finally, the implant contact area after a combinatorial treatment with rhBMP-2 and sEV (79.87% ± 11.86%) was significantly increased when compared to the sEV-treated group (*p* = 0.011), but not in comparison to the Alg only treated group (81.35% ± 17.68%). BIR values determined for the distal implants were again highly heterogeneous with no significant differences between any of the treatment groups (Fig. 2h).

### Biomechanical evaluation

The following specimens were excluded from the biomechanical evaluation due to fractures occurring at the diaphysis during the pull-out test. These exclusions apply to individual samples rather than the entire group: 1x “Alg only-proximal”; 1x “rhBMP-2-proximal”; 1x “rhBMP-2-distal”. Further, one sample of the sEV-distal group was also excluded, as it was not included in the µCT analysis due to improper implant positioning.

The maximum pull-out force (Fmax) and the stiffness were again evaluated separately for the proximal and distal implants (Fig. 3a/b and methods section). As for the volumetric data, significantly different biomechanical values were only determined for the proximal anatomical region (Fig. 3c-f). The rhBMP-2 treatment group showed the highest mean Fmax (78.11 ± 18.66 N), which was significantly greater when compared to the sEV-treated group (53.36 ± 6.669 N, *p* = 0.0043). Comparing the rhBMP-2 treated group and the Alg only group (53.11 ± 20.13 N), there was a tendency for an increased pull-out force after application of rhBMP-2 (*p* = 0.051). For none of the other pairwise comparisons significant differences where determined (Fig. 3c). Stiffness values were again the greatest for the rhBMP-2-treated samples (287.2 ± 84.87 N/mm), which was significantly greater compared to the Alg only group (129.3 ± 29.02 N/mm; *p* = 0.0047). Finally, a significant difference was determined between the rhBMP-2/sEV-treated group (247.2 ± 107.0 N/mm) and the Alg only group (*p* = 0.0379).

### Comparison of proximal and distal implants

**Fig. 4:**
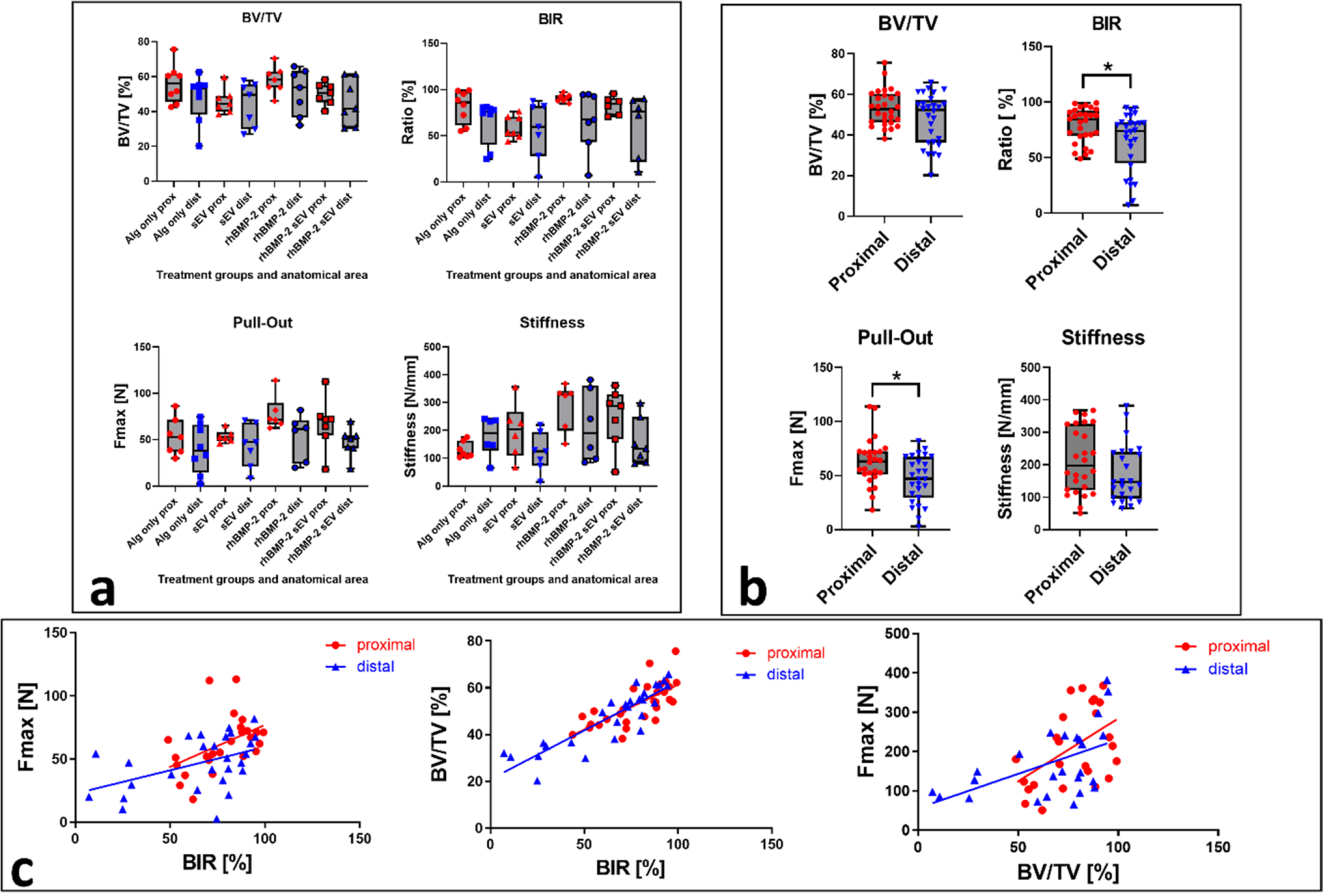
Differences in outcome values determined for proximal and distal implant samples. **a** Representation of the proximal and distal implants regarding BIR, BV/TV, pull-out strength, and stiffness. **b** Cumulative comparison of all outcome measures determined for proximal and distal implants, revealing signficant differences for the BIR (%) and maximum pull-out force (Fmax; N). *p < 0.05 **c** Linear regression analyses for proximal (red) and distal (blue) samples demonstrating a significant correlation between Fmax (N) and BIR (%) (Proximal: R^2^: 0.2197, p-value: 0.0157. Distal: R^2^: 0.2131, p-value: 0.0153.), BIR (%) and Bone Volume/Tissue Volume (BV/TV; %) (Proximal: R^2^: 0.5038, p-value: < 0.0001. Distal: R^2^: 0.7777, *p*-value: < 0.0001.), and Fmax (N) and BV/TV (%) (Proximal: R^2^: 0.2404, p-value: 0.0110. Distal: R^2^: 0.2694, p-value: 0.0078). *p < 0.05

A direct comparison of the individual values (BV/TV, BIR, Fmax, stiffness) determined for the proximal and distal implants within the various treatment groups showed no significant differences, most likely due to the highly heterogeneous results determined for the distal implants (Fig. 4a). While no significant differences were observed between the individual treatment groups when comparing proximal and distal implants, a cumulative analysis across all treatment groups revealed a significant difference between the proximal and distal implant sites in terms of maximum pull-out force (*p* = 0.017) and BIR (*p* = 0.03;). BV/TV (*p* = 0.23) and stiffness (*p* = 0.15) values were comparable between both anatomical regions (Fig. 4b).

Significant linear correlations were identified between radiographic and biomechanical parameters in both the proximal and distal regions. For the relationship between bone-implant ratio (BIR, %) and maximum force (Fmax, N), a moderate positive correlation was observed proximally (R^2^ = 0.2197, slope = 0.6628 ± 0.2550, *p* = 0.0157; Y = 0.6628 × X + 10.63) and distally (R^2^ = 0.2131, slope = 0.3684 ± 0.1416, *p* = 0.0153; Y = 0.3684 × X + 22.60). A stronger correlation was found between BIR (%) and bone volume fraction (BV/TV, %), with R^2^ = 0.5038 and slope = 0.3849 (*p* < 0.0001; Y = 0.3849 × X + 22.86) in the proximal region and R^2^ = 0.7777 and slope = 0.4179 (*p* < 0.0001; Y = 0.4179 × X + 20.86) in the distal region. Furthermore, BV/TV (%) was significantly correlated with Fmax (N), showing R^2^ = 0.2404 and slope = 1.159 (*p* = 0.0110; Y = 3.194 × X – 35.26) proximally, and R^2^ = 0.2694 and slope = 0.5981 (*p* = 0.0078; Y = 1.742 × X + 56.26) distally. These findings indicate that higher bone volume fraction and BIR are associated with increased mechanical stability at both measurement levels.

### Descriptive histology

**Fig. 5:**
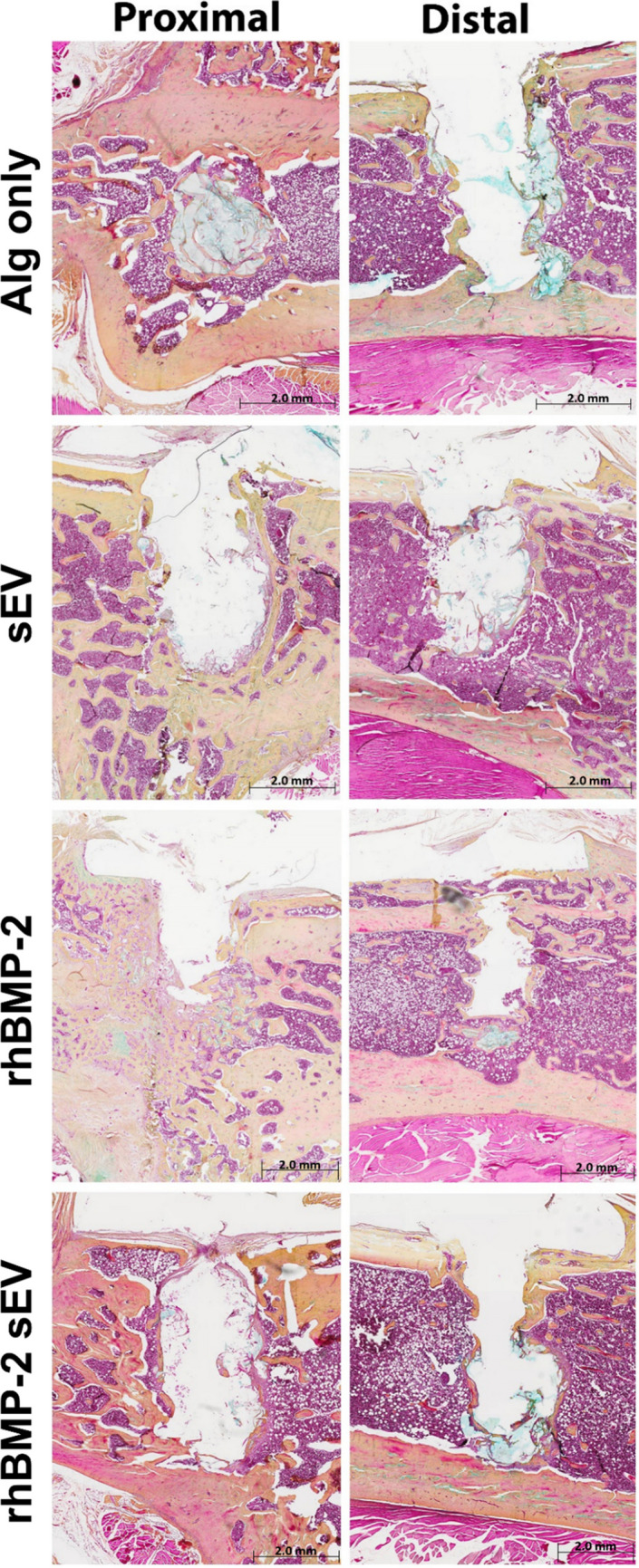
Representative images of Movat pentachrome-stained histological sections of tissue samples harvested 6 weeks after implantation. Overall more abundant new bone formation for the proximal implant sites was observed, whereas the distal implants were only marginally lined with bony tissue. Overall, most abundant bone formation was observed for the proximal implant sites treated with rhBMP-2 (arrows). Remnants of the alginate hydrogel were mostly present for the Alg only group 6 weeks post-surgery (asterisk)

For the drill holes treated with alginate only, the alginate hydrogel was clearly evident within the defect 6 weeks postoperatively (Fig. 5). For proximal implants, formation of new, woven bone was mostly evident for the defects treated with rhBMP-2. In contrast, treatment with sEVs only or a combination of rhBMP-2 and sEVs resulted in less abundant bone formation. Distal implants, regardless of the treatment, where intermittently lined by thin bone.

## Discussion

Inadequate osseointegration of osteosynthesis material is a challenge in surgical fracture management. Poor osseointegration leads to reduced stability and thus to an increased rate of implant loosening, delayed unions and formation of pseudoarthroses, which require revision surgery. In addition, also minor loosening of the implant leads to suboptimal fracture reduction and thus to poorer treatment outcomes [32, 33]. Therefore, external stabilizers such as external fixators, casts, splints, or postoperative partial weight-bearing are often necessary to increase primary stability [34]. This leads to further limitation of the patient, delayed mobilization and joint stiffness. An alternative solution is to fit e.g. an additive osteosynthesis plate. Although this generally increases stability, it requires a more extensive or an additional surgical approach and adequate soft tissue quality [35, 36]. Many studies show a positive effect of immediate full load bearing or rapid increase of allowed load on the final clinical outcome [37]. The treatment goal should therefore be to rapidly achieve a high level of stability with the accompanying permitted full weight-bearing or at least orthosis-free follow-up treatment.

The initial aim of this investigation was to evaluate the potential of human umbilical cord MSC-derived small extracellular vesicles in combination with a low dose of rhBMP-2 to enhance the integration of a screw implant. The newly developed surgical model also allowed the evaluation of screw osseointegration at two different anatomical regions after stabilization using an angular-stable plate. Surprisingly, the functional outcomes were significantly different when comparing the results for the proximal and distal screw implants. Since the therapeutic regimen was the same for both implants within one animal, the results suggest that functional osseointegration is critically influenced by the anatomical location, as improved outcomes after local application of a low dose of rhBMP-2 was only evident for the proximal implants, whereas for the distal implants no significant difference for any of the outcome parameters investigated was observed. Generally, bone tissue adapts to changes in tissue strain, shear stress, as well as fluid flow and osseointegration is significantly influenced by mechanical stresses [38]. In addition, bone growth is also sensitive to fluid pressure experienced by the tissue during mechanical stresses. In line with this, a rat model of dynamic skeletal muscle stimulation demonstrated that the rate of bone adaptation differed across various regions of the distal femur, with a more pronounced response observed near the marrow cavity and a diminished effect near the growth plate [39]. Therefore, it seems plausible that the different outcomes observed between the proximal and distal screw implants observed in this study is the consequence of different mechanical stresses occurring during loading.

To the best of our knowledge, this is the first study to examine the effect of naïve hUC-MSC-derived sEVs on implant osseointegration. Contrary to expectations, hUC-MSC-sEVs alone did not improve osseointegration within 6 weeks after surgery. This finding contrasts with prior reports showing that MSC-EVs can enhance bone repair and implant integration. For example, Fan et al. demonstrated improved osseointegration of an EV-functionalized PEEK implant in a rat femoral defect model, although biomechanical testing was not included in that study [40]. Likewise, Maiborodin et al. reported increased bone density in rabbit tibia following sEV treatment based on densitometry and descriptive histology [22]. Notably, both studies used sEVs from bone marrow–derived MSCs, which may indicate a source-dependent effect on osseointegration. Further, our study employed a xenogeneic approach administering human UC-MSC-sEVs in rat osseointegration model, whereas Fan et al. used a syngeneic system. Although a xenogeneic setting could theoretically dampen EV efficacy, available data do not support immune rejection as the main cause. A recent study showed that systemic administration of human MSC-EVs did not trigger a humoral immune response in a mouse model of renal artery stenosis [41]. In addition, our own published work and that of others demonstrate that hUC-MSC-EVs are well tolerated in multiple small and large animal models, including fracture repair, further supporting the notion that a single application of EVs overall elicits a negligible immunogenic response [28–30, 42, 43].

The potential of rhBMP-2 to enhance osseointegration remains a topic of debate. Although some promising results have been observed, these findings are primarily based on preclinical studies [44–46], and the optimal dosage remains to be determined. Overall, a low dose of 1.5 µg of rhBMP-2 was not expected to result in optimal bone formation or integration based on previous studies demonstrating that lower doses, such as 1–1.5 µg, were insufficient for bone healing in critical-sized defects [47, 48]. Yasko et al. further demonstrated that a dose of 1.4 µg of rhBMP-2 was insufficient to promote robust consolidation of a 5 mm rat osteotomy, while a higher dose of 11 µg resulted in a strong union [49]. Similarly, in a recent study, we found that a 1.5 µg dose of rhBMP-2 was insufficient to repair a metaphyseal femoral defect in an osteoporotic rat model [28]. However, in the present study, treatment with 1.5 µg of rhBMP-2 led to improvements in functional osseointegration at the proximal implantation site. Notably, co-application of 1.5 µg rhBMP-2 with 2 × 10⁹ hUC-MSC-sEVs did not produce additive or synergistic effects on implant osseointegration. This contrasts with our previous work, in which combined application improved healing of a segmental metaphyseal defect [28]. Although the present sEV preparation differed in total protein content and exhibited a modestly smaller mean particle size compared to the preparation used in the previous study, these differences do not readily explain the absence of a synergistic response to the low-dose BMP-2. The underlying reason for this discrepancy remains unresolved and warrants further investigation. However, extrapolating findings from a segmental defect model to an osseointegration model, presents challenges due to fundamental differences in bone healing dynamics between these models. The regeneration of a segmental defect requires substantial bone formation for bridging the defect, whereas osseointegration relies on the direct apposition of bone to an implant surface, which primarily involves bone remodeling rather than extensive new bone formation. Furthermore, the screw implants in the present study were placed in the dense cortical bone of the femoral diaphysis of healthy animals, whereas the metaphyseal regions, especially in osteoporotic bone, consist of less dense and structurally weaker trabecular bone.

Extracellular vesicles (EVs) have been extensively evaluated in preclinical models for their potential to enhance bone repair [50]. They have demonstrated the ability to support bone regeneration through multiple mechanisms, such as promoting bone mineral deposition, stimulating blood vessel formation, modulating the immune response, and driving the differentiation of osteoblasts and osteoclasts [51]. In addition to naïve EVs, particularly those derived from mesenchymal stromal cells (MSCs), several studies have explored engineered EVs to improve their pro-osteogenic properties. For instance, the overexpression of microRNA miR-101 in MSCs promoted osteogenic differentiation *in vitro* [52], or miR-375-enriched EVs developed by overexpressing this microRNA in human adipose MSCs enhanced osteogenic differentiation in human bone marrow MSCs (bMSCs) and promoted bone regeneration in a rat calvarial defect model [53]. The overexpression of pro-regenerative proteins in MSCs has also been explored, with HIF-1α-overexpressing EVs from bone marrow MSCs enhancing both osteogenesis and angiogenesis, and improving bone regeneration in a rabbit glucocorticoid-induced osteonecrosis model [54]. Similarly, EVs from BMP-2 overexpressing MSCs promoted osteogenesis in rat calvaria defects, however likely through modulating microRNAs rather than by BMP-2 itself [55]. Another study demonstrated that EVs derived from human adipose-derived stromal cells engineered to overexpress BMP-2 and VEGF-A improved bone repair in a rat femur drillhole defect model [56]. Despite the potential of engineered EVs, challenges such as transduction efficiency, high costs, and safety concerns, remain barriers to clinical translation and require additional testing when compared to the production from non-genetically modified cells.

The combination of EVs with biomaterials like bioceramics, polymers, and composite scaffolds also shows promise in enhancing bone repair, with hydrogels such as hyaluronic acid (HA) and alginate aiding in the delivery of EVs [57, 58]. Hydrogels based on functionalized polysaccharides [59] or based on a combination of thiol-modified hyaluronan, heparin, and denatured collagen [53] have been explored for the localized delivery of sEVs in preclinical models, with promising results in promoting bone repair. In another study, a combination of alginate hydrogel and polycaprolactone (PCL) scaffolds loaded with BM-MSC-EVs stimulated increased bone formation and vascularization when subcutaneously implanted in nude mice [60]. Together, advances in extracellular vesicle research and production, particularly when combined with smart nanomaterials and scaffolds, present a promising acellular alternative to traditional cell-based therapies.

The following limitations of this study need to be acknowledged. The decision to locally deliver sEVs, with or without rhBMP-2, was made to maximize their concentration at the defect site, considering the short half-life of BMP-2 and the rapid uptake kinetics of sEVs. While a release kinetics study could have provided more insights into the availability and distribution of BMP-2 and/or sEVs administered to the defect site, our study was primarily designed to evaluate the overall effectiveness and observable outcomes in a relevant animal model. Further, based on prior research it is unlikely that the alginate/CaCl_2_ hydrogel at the concentration used in this study (1.5%) would have resulted in a significant retention of rhBMP-2 and/or hUC-MSC-sEVs beyond the expected rate through diffusion and hydrogel disintegration. This is supported by studies showing that crosslinked alginate/CaCl_2_ microspheres release nearly 100% of their protein load (BSA) within 6 h [61]. Finally, our study aimed to evaluate a delivery method for naïve EVs, with potential for clinical translation. While utilizing genetically modified producer cell lines, engineered EVs, or more complex delivery systems could have potentially resulted in improved outcomes, these approaches would introduce significant regulatory and technical hurdles, complicating clinical translation.

## Conclusion

The findings of this study demonstrate that naïve hUC-MSC-sEVs did not enhance implant osseointegration and even slightly reduced the amount of newly formed bone around the screw implant. Only a low dose of rhBMP-2 (1.5 µg) effectively promoted implant ingrowth, with no evident additive or synergistic effect when combined with hUC-MSC-sEVs. Interestingly, the pro-osteogenic effect of the low-dose rhBMP-2 was observed exclusively at the proximal aspect of the rat femur, suggesting that the anatomical region significantly influences implant osseointegration. This highlights the need to consider anatomical location in future studies evaluating the osteoinductive and osteoconductive properties of new implant materials or treatments.

## Supplementary Information

Below is the link to the electronic supplementary material.

Supplementary file1 (DOCX 222 KB)

## Data Availability

For data and material requests, kindly contact the corresponding author.

## Declarations

### Declaration of AI and AI-assisted technologies in the writing process

AI was used to rephrase individual sentences.
