# Supplementary material for: Anatomical implant region – a critical determinant for the osteogenic potency of small extracellular vesicles and rhBMP-2
Source: Eur J Trauma Emerg Surg. 2025 Dec 18;51(1):359. doi: 10.1007/s00068-025-03036-w (PMC12715041; doi:10.1007/s00068-025-03036-w)
Supplement: Supplementary file 1 — Supplementary file1 (DOCX 222 KB) [file 68_2025_3036_MOESM1_ESM.docx]

**
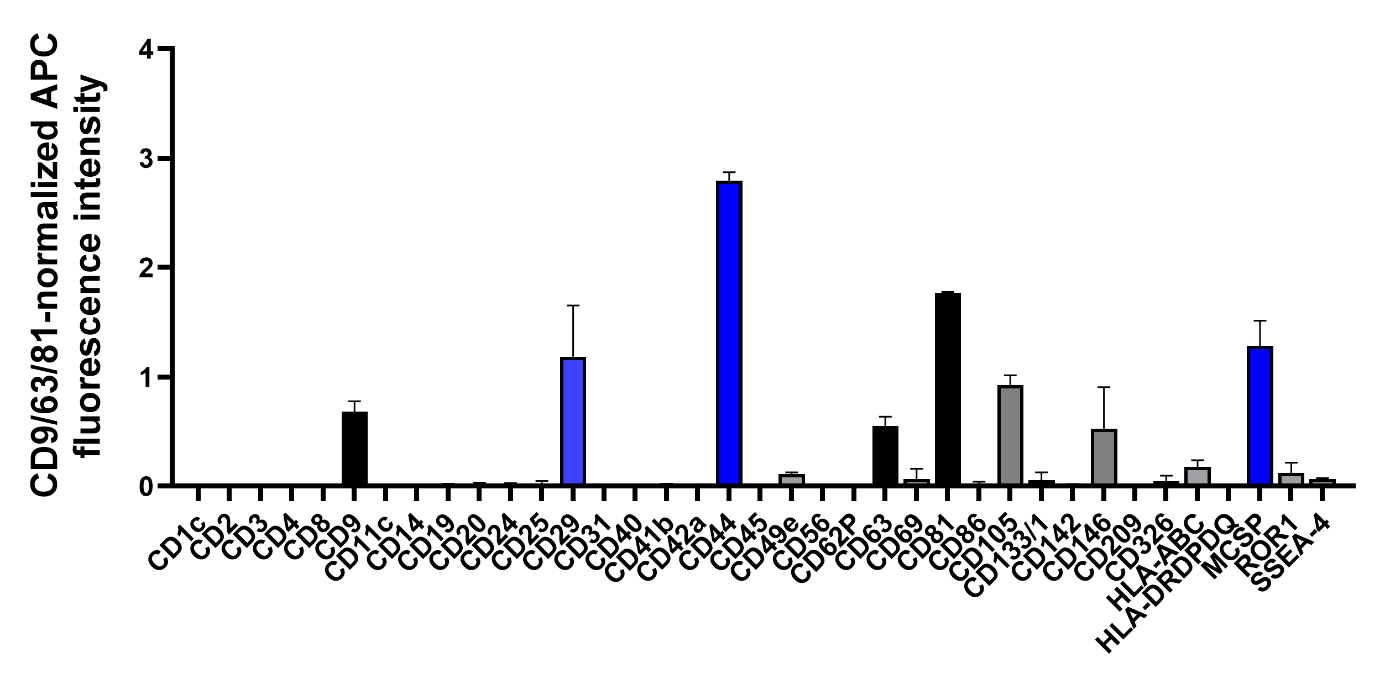
**

**Suppl. Fig. 1.** MACSPlex surface profiling of hUC-MSC-sEVs. Bead-based flow analysis confirms the presence of canonical EV tetraspanins (CD9, CD63, CD81; black bars) and robust expression of markers typically associated with MSC-derived EVs, including CD29, CD44, and melanoma-associated chondroitin sulfate proteoglycan (blue bars). Shown are normalized mean fluorescent intensity values +1SD.
